# Leadership in Public Health: Opportunities for Young Generations Within Scientific Associations and the Experience of the “Academy of Young Leaders”

**DOI:** 10.3389/fpubh.2019.00378

**Published:** 2019-12-17

**Authors:** Vincenza Gianfredi, Federica Balzarini, Marco Gola, Sveva Mangano, Lucia Federica Carpagnano, Maria Eugenia Colucci, Leandro Gentile, Antonio Piscitelli, Filippo Quattrone, Stefania Scuri, Lorenzo Giovanni Mantovani, Francesco Auxilia, Silvana Castaldi, Stefano Capolongo, Gabriele Pelissero, Anna Odone, Carlo Signorelli

**Affiliations:** ^1^Post-graduate School of Hygiene and Preventive Medicine, Department of Experimental Medicine, University of Perugia, Perugia, Italy; ^2^Post-graduate School of Hygiene and Preventive Medicine, University Vita-Salute San Raffaele, Milan, Italy; ^3^Department of Architecture, Built Environment and Construction Engineering, Politecnico di Milano, Milan, Italy; ^4^Post-graduate School of Hygiene and Preventive Medicine, University of Milano-Bicocca, Milan, Italy; ^5^Post-graduate School of Hygiene and Preventive Medicine, University of Bari “A. Moro”, Bari, Italy; ^6^Department of Medicine and Surgery, University of Parma, Parma, Italy; ^7^IRCCS Policlinic San Matteo of Pavia, Pavia, Italy; ^8^Post-graduate School of Hygiene and Preventive Medicine, University of Milan, Milan, Italy; ^9^Post-graduate School of Hygiene and Preventive Medicine, University of Pisa, Pisa, Italy; ^10^School of Pharmacological Sciences and Health Products, University of Camerino, Camerino, Italy; ^11^Department of Public Health, Experimental and Forensic Medicine, University of Pavia, Pavia, Italy

**Keywords:** leadership, public health, education, NGOs, academies, Italy

## Abstract

This paper outlines the characteristics of scientific leadership and the role of Scientific Associations with their specific activities. The recent activities of the Lombard Academy of Public Health are subsequently described, including the creation, in 2019, of the Academy of young leaders in public health. Comparing to other sectors, scientific leadership dynamics take into consideration different aspects. Besides awards (Nobel Prize or several other) and prestigious affiliations, eventual indicators might be academic roles, fundraising abilities, relevant positions among scientific associations, editors of prestigious journals or editorial series and, more recently, high bibliometric indicators. The peculiar topics of public health encompass interactions with institutions, authorities, politicians, involved in different levels in health policies. Recently, in Italy, the Ministry of Health has identified parameters to be accreditated as a scientific and technical association. The role of SItI (Italian Society of Hygiene), EUPHA, ASPHER, and WFPHA appears relevant in PH, in national and international contexts, with Italian praiseworthy members constantly achieving leading roles. Considering that few training opportunities aimed to improve research and leadership skills are available, *Accademia Lombarda di Sanità Pubblica* (ALSP) designed the AYLPH (Academy of Young Leaders in Public Health) program. AYLPH program is a 1-year training to shape leadership skills among young professionals. A set of didactic, theoretical and practical methods was offered and evaluated.

## Introduction

Leadership is an innate aspect of human behavior and, when lacking, progress in society, at community, national or individual level cannot be achieved. Sectors where providing leadership are several, ranging from politics, trade unions, business, military, sports and science (1). However, regardless of the sector, some required qualities are shared: any leader needs to have a vision, to be innovative, ready to take (controlled) risks, curious, flexible but determined and—lastly yet importantly—brave (2). Particularly focusing on scientific leadership, some aspects are partly different compared to other sectors. Indeed, besides awards and prestigious affiliations, other leadership indicators may be academic roles, fundraising abilities, positions within scientific associations, or scientific journals editorial boards and, more recently, in Italy, the updated bibliometric indicators of the National Agency for the Evaluation of Universities and Research Institutes (ANVUR).

Generally speaking, and considering Italian situation, in the current health care system, undermined by the progressive aging population (3), distrust in institutions, antimicrobial resistance and vaccine hesitancy (4, 5), lack of equity and unhealthy lifestyles (6, 7), there is an urgent need of powerful and effective leaders. In this context, public health is emerging from a behind-the-scenes discipline to a trans-discipline that integrates theoretical concepts with health-care assistance (8). Public health leaders play a crucial role in ensuring population health and well-being through prevention and health promotion (9, 10), healthcare policy and management (11), promoting collaboration and networking (12), and through a more effective decision making process (13). Capacity building is the key to success, as reliable and accountable communicators may build trust (14). Health leaders take actions that shape the future of public health and advocate to introduce new health policies (15). Professional development of public health leaders requires competency-based education to help them develop skills to address complex and evolving demands of health care systems (16). This is in line with the WHO (World Health Organization) approach, establishing “Strategic leadership for health” among the ten key areas of public health practice (17).

Nowadays, leaders are not always individuals: they may also be organizations or movements that exert advocacy on politicians and policy-makers. Among them, civil society representatives including unions, consumers' and patients' associations, professional bodies, Non-Governmental Organizations (NGOs) and volunteers' association, and particularly scientific associations play a crucial role (18). In fact, they are involved in the promotion of health through scientific research, communication to the public and to health professions, and in advocacy toward policy makers and institutions (19). Scientific associations are NGOs created to foster informative and educational exchange among members (networking), offering various types of activities, such as conferences, symposia, congresses, work and study groups, and promoting permanent and innovative communication media. These roles are formally recognized to scientific societies by specific laws: they should identify best practices in health, provide technical support to health policy makers, and advocate all the stakeholders with updated scientific evidence. More recently, the Italian Minister of Health has identified parameters to make scientific societies lawfully responsible of guidelines and best practices. The Italian legislative framework is synthesized in Table 1. In the public health sector, the Italian Society of Hygiene, Preventive Medicine and Public Health (SItI) has a high impact at national level—now flanked by two public health academies, the *Accademia Lombarda* and *Accademia Romana*—whilst the EUPHA^1^, ASPHER^2^, and WFPHA^3^ are the most relevant public health scientific society at international level, with Italian praiseworthy members constantly achieving leading roles (20, 21).

**Table 1:** Italian legislative framework that formally recognized the roles of scientific societies.

**Regulation**	
*Art. 16*-ter Legislative Decree 502/92 modified by Legislative Decree 229/99	The National Commission for Continuing Education shall also define the requirements for the accreditation of scientific societies as well as public and private entities carrying out training activities and shall verify the subsistence of requirements themselves.
The National Health Plan 2003–2005	It recognizes the role of scientific societies as guarantors not only of the soundness of the scientific bases of training events, but also of the pedagogical quality and their effectiveness
Ministerial Decree 31 May 2004	National Commission for Scientific Training's accreditation criteria
Art 3 Law 8 November 2012, n. 189, Decree Law 13 September 2012, n. 158	If, during the execution of its activity, the health professional follows the guidelines and best practices, recognized by the scientific community, then he/she is not subject to criminal liability for minor misconduct. However, in such cases, the obligation laid down in Article n. 2043 of the Civil Code shall remain unchanged. “The judge, in determining damages, shall take due account of the conduct referred to in the first sentence”
Ministerial Decree 2 August 2017	It establishes the list of scientific societies and technical-scientific associations of health professions—issued for implementation of art. 5 of the Law n. 24 of 8 March 2017 (Gelli Law), on “Provisions on the safety of care and of the assisted person, as well as on the professional responsibility of health professionals”
	For the purpose of inclusion in the list, scientific societies and technical-scientific associations of the health professions shall meet the following requirements: (a) be relevant at national level […] (b) to be representative of at least 30% of professionals in the sector and 12% of general practitioners (c) deed of incorporation drawn up by public deed and bylaws

Nowadays, scientific societies are suffering from aging of members, real or potential conflicts of interest, lack of remuneration for active members and possible internal conflicts; however, scientific societies are still pivotal to shape the future of public health (22). In order to go beyond this momentary crisis, scientific societies should be aware that they are representing the core values of the discipline, work hard to foster cultural and moral credibility, and reduce or avoid conflicts of interest. Moreover, they should advocate in terms of dissemination of corporate vision, promote mutual exchanges between leaders and members in order to solve organizational and professional problems; develop new forms of networking among members, providing an education “close to members” addressing needs, training design, outcome verification; and lastly they should invent role for “emeritus” and incentives for young members (18, 23).

In this perspective, the *Accademia Lombarda di Sanità Pubblica* (ALSP, Lombard Academy of Public Health), a non-profit association founded in 2017, aims to promote progress in public health through the involvement of experts engaged in many areas of public health (e.g., hygiene, epidemiology, prevention, environment, health management, health care, management, law, and health economics), promoted the AYLPH (Academy of Young Leaders in Public Health). The AYLPH may be assumed as a platform to discuss about health issues, through different perspectives and backgrounds, and the final message can be spread, not just delivered, but rather shared with the general population. The important attribute is the creation of a common sense of membership, with this principle of leadership fully reflected in the health promotion notion of empowerment: enabling people to improve their health (24, 25). Moreover, the requirements of future leaders certainly cannot exclude emotional skills and intercultural communication competencies, in order to lead effectively in multinational organizations. Collaboration and shared responsibilities can facilitate the decision-making and show cohesion, which surely influence the perception of the institutions the leader represents (17). Taking into account these essential requirements, the experience of the AYLPH represented an opportunity to establish discussions, expand a network, sharing activities in the international events, but always bringing back the message to each local social and scientific community, where each of the participant belonged to.

### Aim

Considering that few training opportunities aimed to improve research and leadership skills in public health are available, ALSP designed the AYLPH program. The program aimed to build the leadership skills and to educate young public health professionals to be the future workforce trained for research, and as well respond to public health needs. The aim of this paper is to describe the conceptual framework behind the AYLPH program, the implementation process, and the preliminary results obtained.

## Pedagogical Framework

### Conceptual Framework

The AYLPH program is designed and promoted by the ALSP; however, the steering committee members were the full professors in Hygiene and Preventive Medicine of Lombardy region in Italy. Overall, five academic departments were involved in the project (Università degli Studi di Pavia, Università degli Studi di Milano, Università degli Studi di Milano-Bicocca, Università Vita-Salute San Raffaele, and Politecnico di Milano). Each department was in charge of organizing one theme-based module supervised by an acknowledged expert. AYLPH program was structured on evidence-based public health leadership competency framework (26), focusing on acquiring abilities in synthetizing and analyzing available evidence, with lenses and values of public health. More in details, the program aimed to develop the following competencies: build capacity to make decisions based on the best evidence, stimulate emotional intelligence, networking, build up own vision, using available knowledge, communicate findings and apply scientific evidence to public health issues, improve the multidisciplinary and interdisciplinary approach in public health. Furthermore, the innovations of the program are mainly located in (i) the shifting from classical frontal lessons to interactive and experience-based meetings, (ii) the interdisciplinary learning, and (iii) the international health interests.

### Selection Process

The training program was advertised internally and externally by mail and the web-page of ALSP (27). The recruitment was opened to all young members of the Academy, regardless of their region of origin. The call clearly defined the professional and research line proposed. Subjects interested in participating were asked to submit the application trough the web-page of Academy, with a cover letter and Curriculum Vitae.

Criteria for eligibility were: to be a young member of the Academy with previous research experiences, good level of English, and previous training in hygiene institutions and departments. Each item was scored from 0 to 5. The Academy members committee (full professors in Hygiene and Preventive Medicine, directors of Post-Graduate School) independently screened the applications. The selection system aimed to identify highly motivated candidates for leadership careers in public health, already undergoing professional and research careers. Furthermore, another objective of the selection process was to identify young professionals with highly diversified education backgrounds, also without a specific health professional education, but sharing the public health vision. To promote equity, the gender and geographic representativeness has been also considered, taking into account the expertise and the motivation of the candidates.

## Learning Environment

### Implementation of the Training Program

AYLPH program is developed within 1-year time-frame, and it is a training program aimed to promote operational research and leadership skills among young professionals. During the year, a set of didactic, theoretical and practical methods in the frame of public health leadership was offered. In particular, the first track consisted of a 2-days immersive winter team-building course, during which a self-assessment evaluation was performed, in order to be aware of personal skills, knowledge, motivations, background and foster emotional intelligence. Moreover, during the first track, participants had the opportunity to meet some national and international leaders in public health. The meeting was an informal but very interactive discussion between participants and leaders.

The second track was designed to improve literature search abilities, develop mentorship skills and formulate research questions relevant for public health. The intensive and comprehensive systematic revision course took place at the University Vita-Salute San Raffaele in Milan and it was hosted by the local postgraduate school of hygiene and preventive medicine. The course was opened not only to AYLPH participants, but also to other medical doctors, residents in public health. During this course theoretical, methodological and practical knowledge was delivered; however, academic members encouraged participants to formulate new research questions using systematic reviews as study design. Furthermore, academic members elicited students and participants to set small research groups headed by at least one of the AYLPH members, aimed to implement the research projects. This group-work approach allowed participants to train their leadership, through coaching the other colleagues.

The third track consisted in attending national and international conferences that participants could self-opt from an approved list. Participants were encouraged to take part to important conferences in order to get inspired and bring out their own vision.

The last track was a 2-days course on public speaking aimed to improve both verbal and non-verbal communication skills, to teach the new technology and traditional media use, and to target the communication considering the audience. The course took place at the University of Pisa and was hosted by the local postgraduate school of hygiene and preventive medicine.

### Deliverables

Evaluation method is based on the achieved program outputs and research projects development, number and quality of abstracts submitted to conferences and accepted as poster or oral presentation, and scientific publications obtained by participants. Lastly, a formal evaluation (multiple-choice test) is part of the final assessment of the skills learned by participants.

## Experiences and Results

A total of 18 applications were submitted; among these, 10 participants (six women and four men) with the highest scores were selected. The participants came from 10 different Italian universities and had different backgrounds. Among them, seven were medical doctors, resident in public health, an architect for public health, and two biologists, with a mean age of 34.1 years. Three of them hold a PhD and one of them is a university researcher.

### First Track

The first field-immersion winter track was conducted in a mountain area, hosted in a lodge with few contacts with the external. This first meeting was very helpful to stimulate interactions between participants and faculty members, providing both learning and convivial opportunities. Furthermore, the self-assessment method encouraged the self-awareness of individual and professional limits and strengths, as well as, endorse engagement between participants. After that, a journey statement has been asked to all participants. The statement judged by the committee as the most representative of the activities performed was published on Twitter. Among the international leaders in public health met by the participants, it is worth mentioning the former Italian health minister, the editor-in-chief of one of the top scientific journal in public health, the EUPHA President, the research director of the European Observatory on Health Systems and Policies, and the former president of the Italian National Institute of Public Health. This meeting offered the chance to discuss about ethics in research, new challenges of public health, tips and tricks on publication process, and the important role of advocacy in public health. Lastly, personal and professional experiences were shared.

### Second Track

In the second track, the 10 participants and 14 medical doctors, resident in public health, (voluntary enrolled among the lombardian three schools of Public Health) attended the systematic review seminar. During the outcome-oriented workshop, six research groups were set up, with precise research questions and projects.

### Third Track

In the third track, participants—divided into three groups—attended the General Assembly of the World Health Organization, the Deans' and Directors' Retreat of the ASPHER, and the American Public Health Association meeting (APHA).

### Forth Track

During the fourth track participants learned (i) how to persuasively communicate to public, colleagues, policy makers and other key stakeholders, using evidence; (ii) how to design effective communication campaign; (iii) main differences between traditional and new media. All participants attended all the “tracks” with the exception of the third one, where participants were split between the different activities. Furthermore, participants already achieved some deliverables. Two participants have submitted two abstracts to the Italian national conference of hospital medical managers, and both received the award as second-best poster and second-best communication. Another abstract written by all the participants has been submitted to the Italian national conference of public health. One participant had a speech, as a young professional, in the ASPHER Deans' and Directors' Retreat. The six research projects began in April, and all of them are ongoing. Lastly, the current paper is part of AYLPH program's results. Results from the final evaluation are still in progress.

## Discussion

According to a European survey (28), and a more recent Italian survey (29), public health trainings lack of drawing out leadership qualities. In this regard, creating a program for young professionals highly motivated for leadership careers in public health is a valuable educational goal, especially considering the innovative didactic methods used. In the first AYLPH program, the recruited participants came from five different Italian Regions (30), and it was an added value, highlighting that public health is not a “regional health.” Another strength of the participants' group is the multidisciplinary that enriches the group with a valuable diversity, which in turn contributes to stimulate discussions on public health aspects in a more comprehensive way (31). Indeed, the program committed to train young leaders and to promote researches addressing relevant public health issues, through a competency-based course to help them develop leadership requisites, including emotional intelligence, evidence-based decision, communication, and networking. According to Goleman (32), emotional intelligence is a fundamental component of the leadership course (32), and it is one of the competencies that leader in public health should develop in order to be aware of the inspiring values (33), and being empathetic with citizens, which are both the target population of public health interventions and the actors of the changes too (34).

Whilst, the review projects have given the participants the opportunity to put into practice the acquired competence of both analyzing/summarizing evidence and leadership, by training and mentoring students and other colleagues. Furthermore, these became as well a very important occasion to improve teamwork attitude. Participating in national and international conferences gives the great chance of meeting decision-makers, international leaders, practitioners and peers that contribute in building national/international collaborations, sharing best experiences and grow up with new visions.

Learning rudiments of public speaking is another important aspect for the leaders of the future (35). Public speaking is a powerful and persuasive tool extremely useful in order to motivate and engage citizens to begin and sustain the change (36). Actually, in order to be able to face the challenges of the next century, it is important that citizens increase personal awareness of their important role, as they are the main actors on the stage of their life.

Another strength of this program lies in the stronger emphasis dedicated to personal leadership skills than knowledge. Indeed, the AYLPH program offered the participants not only the theoretical structure, but mainly the opportunity to practice in the field, and to develop the leadership skills in the real world. Through the AYLPH program, participants strengthen their ability in defining relevant research questions, supervising students and colleagues and conducting a research, using available scientific evidence to make decisions, effectively communicating, basing on evidence and considering the audience, recognizing their own limits and strengths, and creating their own vision being as well the change promoter.

Informally, the participants reported a high level of satisfaction mainly due to self-learning coming from teamwork, feedback from peers and from academic members, and from the multidisciplinary and international experiences. The possibility of meeting practitioner experts in the field and researchers has been recognized as another strength. The AYLPH program is an experimental but promising training project to build young leaders and to promote high quality research. In literature, the implementation of other leadership courses is described (34, 37, 38), however, to the best of our knowledge, this is one of the few Italian experiences after the leadership in public health course delivered by the Catholic University of Sacro Cuore in 2017. The national health system extremely needs fresh workforce, highly educated in public health, with high competences in evidence-based practice and with leadership and research skills. Despite the success of the first AYLPH program, still a lot needs to be done. The first lesson learned is the necessity to constantly up-date and rethink the program, considering the rapid evolution of the field. Second, increasing the potential use of new technologies aimed to promote alternative educational methods, which might drop costs through reducing participants' travels, and making the program more sustainable; and probably it could allow delivering courses to a wide number of participants. Lastly, identifying a future financial system able to guarantee a self-funding program would be a great addition.

In conclusion, leadership skills are even more needed in the context of public health in order to both drive the change and increase performance. It is particularly true in the current context, in which community's health is undermined by a sense of distrust in institutions, lack of equity, and unhealthy lifestyles. The new leaders should be able to drive individual, organizational and political change. The traditional public health courses are still not completely able to satisfy the training needs, especially in regards to the practical leadership skills. In this perspective, the AYLPH program aimed to teach new content and mainly new competencies. The preliminary results obtained are encouraging; however, future evaluations are needed in order to corroborate the results.

## Data Availability Statement

The raw data supporting the conclusions of this manuscript will be made available by the authors, without undue reservation, to any qualified researcher.

## Ethics Statement

Ethics approval and written informed consent from the participants of the study were not required in accordance with national legislation and institutional requirements.

## Author Contributions

Manuscript preparation was undertaken by VG and FB. AO and CS reviewed the last version of the article. All authors have contributed to the development of the manuscript and read and approved the final version of the paper.

### Conflict of Interest

The authors declare that the research was conducted in the absence of any commercial or financial relationships that could be construed as a potential conflict of interest.

## Footnotes

^1^European Public Health Association.

^2^Association of Schools of Public Health in the European Region.

^3^World Federation of Public Health Associations.
